# Celastrol impairs tumor growth by modulating the CIP2A-GSK3β-MCL-1 axis in gastric cancer cells

**DOI:** 10.18632/aging.204879

**Published:** 2023-07-19

**Authors:** Jin Wu, Feng Ye, Tao Xu

**Affiliations:** 1 Department of Gastrointestinal Surgery, The Sixth Hospital of Wuhan, Affiliated Hospital of Jianghan University, Wuhan, Hubei, China; 2 Department of Oncology, The Sixth Hospital of Wuhan, Affiliated Hospital of Jianghan University, Wuhan, Hubei, China; 3 Department of Dermatology, The Sixth Hospital of Wuhan, Affiliated Hospital of Jianghan University, Wuhan, Hubei, China

**Keywords:** celastrol, gastric cancer, CIP2A

## Abstract

Background/Aim: High Cancerous Inhibitor of PP2A (CIP2A) expression has been reported in solid and hematologic malignancies and is inversely associated with prognosis in Gastric Cancer, the non-small cell lung cancer, et al. CIP2A can be a drug target for the development of novel anti-gastric cancer agent. Our study was designed to explore the anti-cancer effect of celastrol, a small natural compound, and whether it has an anti-proliferative effect through inducing CIP2A degradation against gastric cancer cells.

Materials and Methods: Employing human gastric cancer cells AGS and BCG-823 cells, the effects of celastrol on cell proliferation, apoptosis and cell cycle was specifically investigated via Annexin V-FITC/PI staining and CCK8 assay. The functional association between celastrol and CIP2A was evaluated by using CIP2A knockdown and overexpression technique. The mechanism of underlying celastrol-triggering anti-gastric cancer effect was detected by real-time PCR and western blot analysis.

Results: Celastrol concentration- and time-dependently induced CIP2A degradation and led to gastric cancer cell apoptosis. More in depth studies revealed specific activation of Protein phosphatase 2A (PP2A)-GSK3β-MCL-1 signaling pathway was involved in pro-apoptosis effect of celastrol, due to celastrol-triggering degradation of CIP2A, which mainly suppressed PP2A activity.

Conclusion: Our findings highlight that celastrol has therapeutic potential via inducing apoptosis of gastric cancer cells.

## INTRODUCTION

Gastric cancer ranks the top four of cancer-related death globally according to the International Agency for Research on Cancer (IARC). In terms of histology, adenocarcinomas is the main type of Gastric cancer, and a small part histologically showed diffuse and intestinal type [1]. Chronic infection was the highest risk for gastric cancer incidence, because high seroprevalence rates for infectious agents were detected in clinical samples. The bacterium Helicobacter pylori and Epstein-Barr virus (EBV) were two main sources of infection. Long-term recurring inflammation could alter cell proliferation, apoptosis and some epigenetic modifications of proto-oncogene and tumor suppressor, eventually leading to oncogenesis [2]. In clinical trials for gastric cancer treatment, target molecular therapy in combination with surgery provides a novel strategy and maybe a therapy tendency for locally advanced disease in the future.

Cancerous inhibitor of protein phosphatase 2A (CIP2A) is a well-characterized human oncoprotein which has been confirmed to possess pro-proliferation and anti-apoptosis ability. Increasing evidence demonstrated CIP2A in ovarian cancer, gastric cancer, colon cancer and non-small-cell lung cancer was overexpressed [3–7]. Furthermore, available clinical trial data indicated CIP2A was a useful predictive marker of poorer prognosis of gastric cancer [7], NSCLC [8], ovarian cancer [9] and chronic myeloid leukemia [10]. In a CIP2A-deficient mouse model, tumorigenesis entered the senescence phase [11]. Mechanistically, the robust tumor-promoting effects of CIP2A overexpression have been linked to inactivate protein phosphatase 2A (PP2A). Inactivation of PP2A selectively dephosphorylated both threonine 308 and serine 473 of Protein Kinase B (Akt) and stabilized c-myc structure in cancer cells [12–14]. Elgendy and colleagues reported that PPA2 activated the downstream glycogen synthase kinase 3β (GSK3β), which led to the decline of pro-survival protein MCL-1 and mediated metformin-induced gastric cancer cell death [15]. E2F1 (an oncogenic transcription factor) mediated transcriptional regulation of CIP2A by attaching to its promoter, and in turn, CIP2A inhibited E2F1 phosphorylation by hindering PP2A. Inactivated p53 promoted CIP2A expression via E2F1-CIP2A feedback, whereas high CIP2A expression in turn relieved E2F1 activity inhibition and senescence induced by p53 [16]. Several compounds, including bortezomib and erlotinib, showed their anticancer effects via this signaling pathway [17, 18]. CIP2A also increased mTORC1-dependent growth signaling and meanwhile mTORC2 inactivation promoted CIP2A binding to PP2A [19]. Accumulating evidence suggests that CIP2A plays an essential role in carcinogenesis. These findings manifested the important role of CIP2A-GSK3β-MCL-1 regulatory axis in gastric cancer.

**Figure 1:**
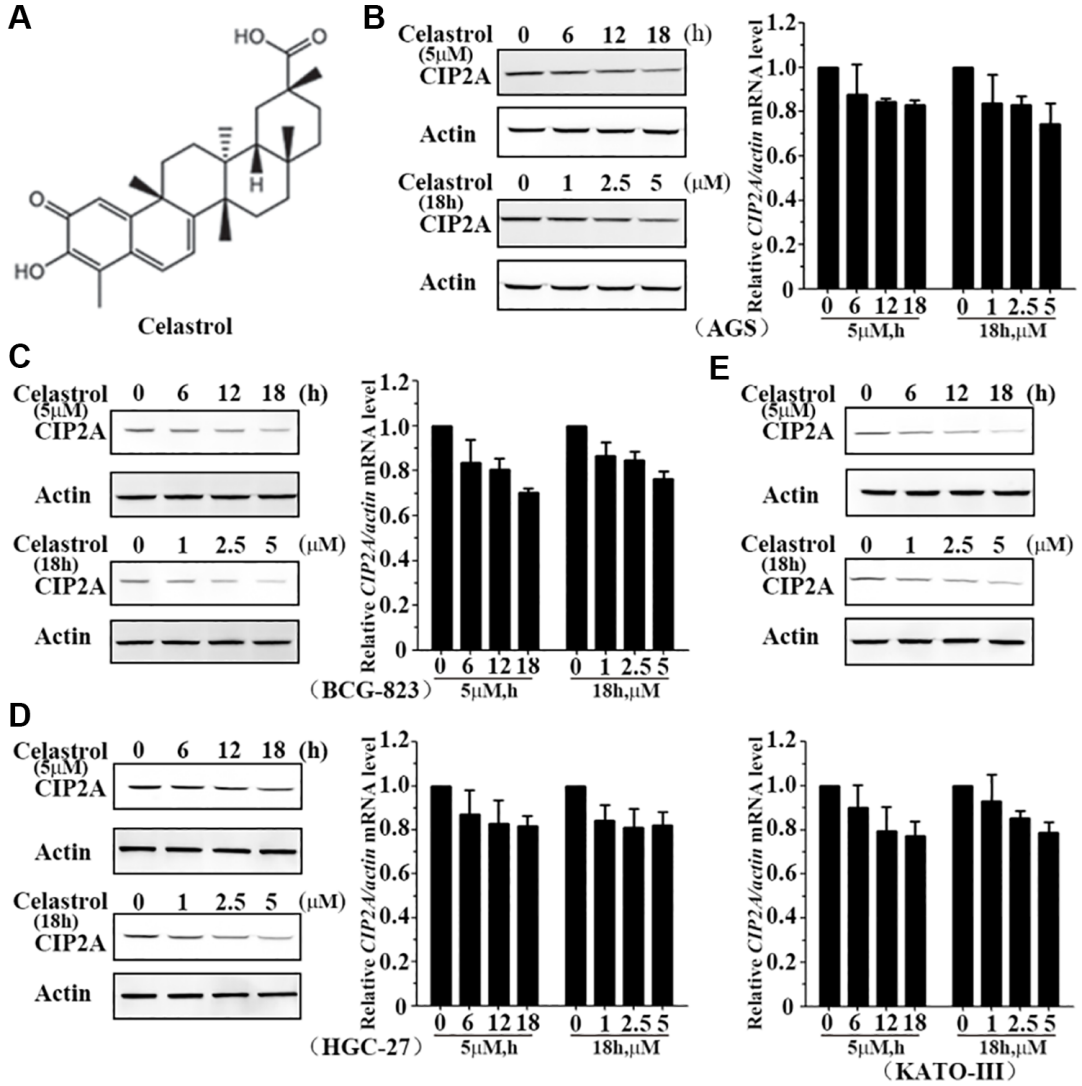
**Celastrol induces degradation of CIP2A.** (**A**) Chemical structure of celastrol. (**B**–**E**) AGS, BCG-823, HGC-27 and KATO-III cells were treated with celastrol at indicated times and concentrations, the expression of CIP2A was detected by mRNA (right or down) and western blotting (left or up) assays.

Celastrol is a plant triterpene (Figure 1A), which derives from the root of traditional medicinal herb thunder god vine [20] and has extensive anti-inflammation and anti-cancer effects. Studies have identified that celastrol affects the tumor growth of gastric cancer, ovarian cancer, pancreatic cancer, glioma, and so on. Celastrol modulate various cellular processes such as cell proliferation, apoptosis, autophagy, and inflammatory response. Mechanistically, celastrol modulates the protein kinase B, vascular endothelial growth factor (VEGF), matrix metalloproteinases (MMPs), production of cytokines and chemokines. However, the underlying mechanism of negative effect role of celastrol on gastric cancer is still unclear. Therefore, in our study, the effects and mechanisms of celastrol on cell proliferation and apoptosis was investigated. We hypothesized that CIP2A is an important target of celastrol and celastrol is capable of inducing apoptosis and growth inhibition through inducing CIP2A degradation mediated proteasome complex and activating its downstream signaling pathway in gastric cancer cell.

## MATERIALS AND METHODS

### Cell culture

The Gastric cancer cell lines AGS, BCG-823, HGC-27 and KATO-III were purchased from the American Tissue Culture Collection. The AGS and KATO-III cells were cultured in Dulbecco’s modified Eagle’s medium (DMEM, Hyclone) and BCG-823 and HGC-27 were cultured in RPMI 1640 (Hyclone) containing 10% fetal bovine serum (FBS, Hyclone), 100 units/ml penicillin and 100 mg/ml streptomycin at 37°C in 5% CO_2_ in a humidified incubator.

### Reagents and antibodies

Celastrol and MG-132 were obtained from Calbiochem (Cat #474790; San Diego, CA, USA). PS-341 was obtained from Millennium Pharmaceuticals (Cambridge, MA, USA). Cycloheximide (CHX) was purchased from Beyotime (Jiangsu, China). These reagents were dissolved in dimethyl sulfoxide (DMSO, Sigma-Aldrich, MO, USA). The primary antibodies were used: anti-CIP2A (HL1925; Santa Cruz Biotechnology, Santa Cruz, CA, USA); anti-PP2A, anti-MCL-1 and anti-Actin (ab137849, ab32087, ab8226; Abcam, Cambridge, MA, UK); anti-GSK3β, anti-BCL-2 and anti-BCL-x_L_ (#9336, #15071, #2764; Cell Signaling Technology, Danvers, MA, USA). Enhanced chemiluminescence kit was obtained from Yeasen Technology (36222ES60; Shanghai, China).

### RNA extraction, reverse transcription-PCR and RT-PCR analysis

Total cellular RNAs were extracted using a Trizol reagent (Invitrogen, Carlsbad, CA, USA) according to the manufacturer’s protocol. 1 μg total RNA were reverse synthesized into cDNAs using random or oligo-dT primers and MMLV reverse transcriptase (AT101; Transgen, Beijing, China). qRT-PCR analysis was subsequently performed with ABI7700 detector (Applied Biosystem, Waltham MA, USA) using the SYBR Premix ExTaq system (RR390Q; Takara, Japan). Sequence of primers used was as follows:

**Table:** 

CIP2A forward	5′-GGGAATTCCCTGATTCCTCTTCA-3′
CIP2A reverse	5′-CCCTCGAGCTAGAAGCTTACTTCCAT-3′
β-actin forward	5′-GTGGGGCGCCCCCAGGCACCA-3′
β-actin reverse	5′-CTCCTTAATGTCACGCACGAT-3′
BCL-2 forward	5′-CATGTGTGTGGAGAGCGTCAA-3′
BCL-2 reverse	5′-GCCGGTTCAGGTACTCAGTCA-3′
BCL-x_L_ forward	5′-TCCTTGTCTACGCTTTCCACG-3′
BCL-x_L_ reverse	5′-GGTCGCATTGTGGCCTTT-3′
MCL-1 forward	5′-CGGTAATCGGACTCAACCTC-3′
MCL-1 reverse	5′-CCTCCTTCTCCGTAGCCAA-3′

The mRNA was normalized to β-actin and calculated using delta method from threshold cycle numbers.

### siRNA and plasmid transfection

The chemical modified siRNAs targeting CIP2A and control were designed and purchased from Shanghai GenePharma. The sequences for CIP2A and control were used as follows: 5′-CUGUGGUUGUGUUUGCACUTT-3′ (CIP2A) and 5′-UUCUCCGAACGUGUCACGUTT-3′ (NC). siRNA transfection into AGS and BCG-823 cells at 100 nM was performed using Lipofectamine 3000 (L3000001; Invitrogen, Carlsbad, CA, USA) according to the manufacturer’s protocols.

The pcDNA3.1-FLAG-CIP2A expression plasmid was constructed and transiently transfected with 2 μg plasmid into AGS and BCG-823 cells using Lipofectamine 3000.

### Western blotting

Cell pellets were extracted with RIPA buffer on ice for 30 min. Protein extracts were normalized and loaded on 10–15% SDS-PAGE and electrophoresed. Gels were transferred onto nitrocellulose membranes followed by blocking with 5% non-fat milk in Tris-buffered saline. The membranes were rinsed and probed with the specific primary and corresponding secondary antibodies. Each Band was visualized by using Luminescent Image Analyzer LSA 4000 (GE, Fairfield, CO, USA).

### Apoptosis analysis

After celastrol or siRNA treatment, the cells were harvested by trypsinization and then subjected to Annexin V-FITC/PI apoptosis detection kit (C1062S; Beyotime, Shanghai, China). Cells were rinsed twice with ice-cold PBS and incubated with FITC-conjugated annexin V for 30 min and then stained with PI added immediately prior to analysis. Apoptosis was determined by flow cytometer (FACSCalibur, by flow cytometer BD Biosciences, San Diego, CA, USA).

### Cell proliferation assay

The anti-proliferative effect of celastrol on AGS and HCG-823 cells was determined by CCK-8 assay according to manufacturer’s protocols. Briefly, cells were plated into 96-well plates with a density of 5 × 10^3^ cells/well, treated indicated time points later with different doses of celastrol. The fresh mixture containing 10% CCK-8 reagent was added to the cells to replace spent medium and incubated at 37°C for 1–4 h, and absorbance was measured at 492 nm using Microplate spectrophotometer.

### Statistical analysis

The quantitative data were presented as the mean ± SD unless noted otherwise. All statistical analyses were two-tailed and computed using GraphPad Prism 5 and SPSS 17.0 software. Student’s *t*-test or Mann-Whitney *U* tests were used to determined statistically significant differences between two or more groups, respectively. *P* values less than 0.05 indicated statistically significant in all cases.

## RESULTS

### CIP2A is rapidly downregulated in cells treated with celastrol

To investigate the effect of celastrol on CIP2A expression level, we treated AGS, BCG-823, HGC-27 and KATO-III cells with celastrol or not and evaluated CIP2A expression level by quantitative real-time PCR and western blot analysis. Results indicated treatment of AGS cells with celastrol at 5 μM for 6–18 h drastically lowered CIP2Aexpression at protein level and became undetectable at 18 h, but very slight change at mRNA level (Figure 1B). Similarly, expression level of CIP2A was reduced response to celastrol treatment at 1–5 μM for 6 h also reduced the (Figure 1B). Furthermore, these similar observations were confirmed in other gastric cancer cells (Figure 1C–1E). In summary, celastrol induced reduction of CIP2A at protein level in a dose- and time-dependent manner, but not at mRNA level.

### Celastrol-induced CIP2A degradation is mediated by the ubiquitin–proteasome pathway

**Figure 2:**
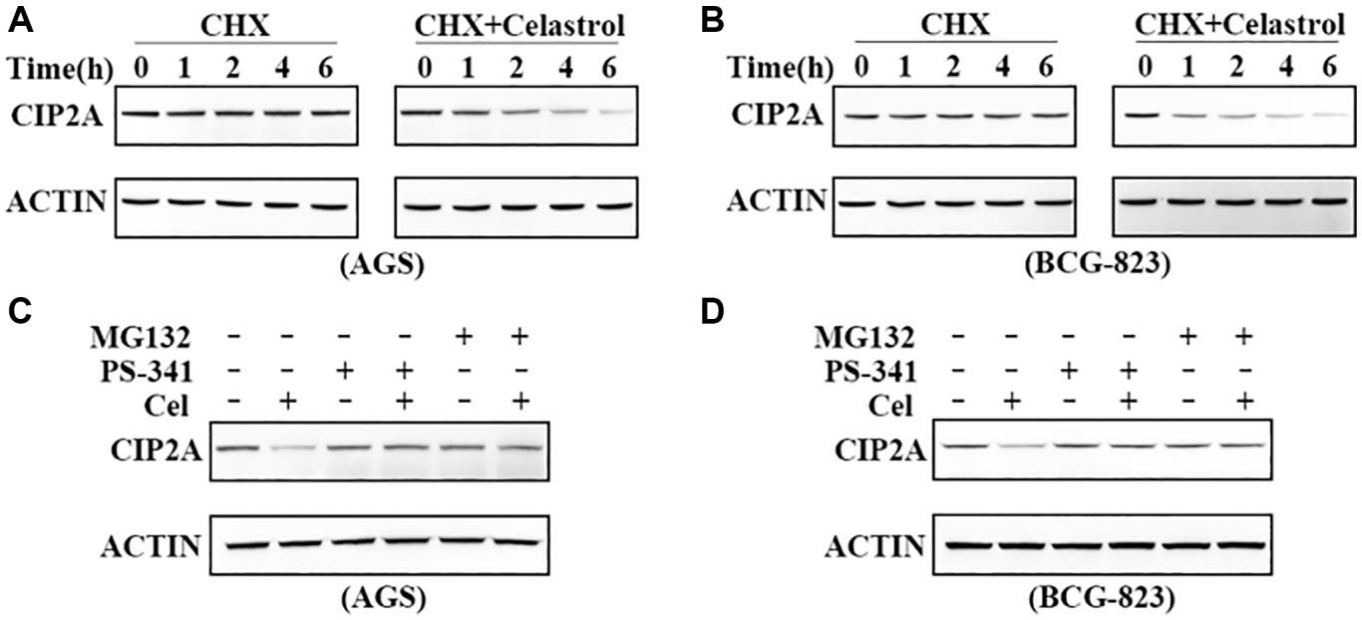
**Celastrol-induced CIP2A degradation is mediated by the ubiquitin–proteasome pathway.** (**A**, **B**) Effects of 50 ug/ml cycloheximide (CHX) alone or in combination with 5 μM celastrol on CIP2A expression were evaluated by western blotting in AGS and BGC-823 cells. (**C**, **D**) Western blotting analyzed CIP2A expression in AGS and BGC-823 cells preincubated with PS-341 (100 nM) and MG-132 (10 μM) for 2 h followed by treatment of celastrol (5 μM) for additional 6 h.

The underlying mechanism of CIP2A reduction is important for celastrol clinical application. CIP2A was downregulated at 2–6 h after celastrol treatment using protein synthesis inhibitor cycloheximide (50 μg/mL) to block de novo protein synthesis, indicating that CIP2A ablation was due to its proteolytic degradation. Of course, only cycloheximide could not reduce CIP2A expression level within 6 h (Figure 2A, 2B).

The ubiquitin-proteasome system is involved in targeted degradation of intracellular short lived proteins in mammalian cytosol and nucleus [21]. To explore and clarify the mechanism of CIP2A degradation, we first detected the effect of classic proteasome inhibitors PS-341 and MG-132 on the degradation of CIP2A. Interestingly, western blot results demonstrated only proteasome inhibitors stimulation could not influence CIP2A stability. However, the phenomenon of celastrol-triggered CIP2A degradation was blocked when these inhibitors were added to the medium with 5 μM celastol within 6 h (Figure 2C, 2D). These observations clearly indicated that ubiquitin-proteasome was the essential pathway of celastrol-triggered CIP2A degradation.

### Celastrol suppresses proliferation and triggers apoptosis of gastric cancer cells

**Figure 3:**
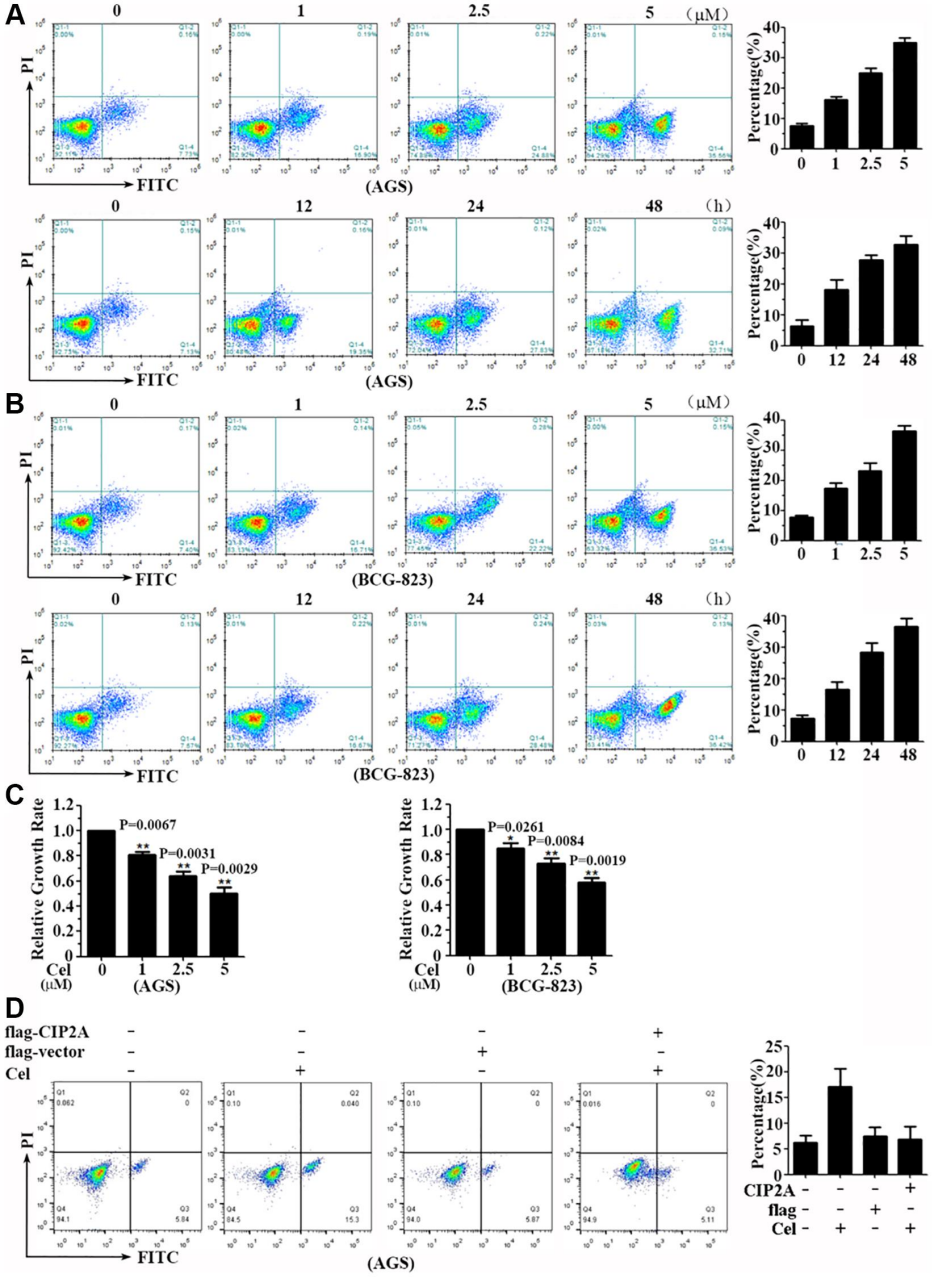
**Celastrol triggers apoptosis of gastric cancer cells.** (**A**, **B**) AGS and BCG-823 cells were treated with various concentrations of celastrol for 24 h or 5 μM celastrol for indicated times, and cell apoptosis was assessed by annxin V-FITC/PI staining and flow cytometry. (**C**) AGS and BCG-823 cells were treated with gradually increasing doses of celastrol for 24 h. The cell viability was measured by CCK8 assay. (**D**) AGS and BCG-823 cells were transfected with CIP2A-flag expression vector for 48 h, followed by celastrol (5 μM) for 6 h, cell apoptosis was assessed by annxin V-FITC/PI staining and flow cytometry.

Our previous studies indicated anti-cancer effect of celastrol was associated with CIP2A reduction. We have known CIP2A was a direct target of celastrol. Next, our research focused on the anti-gastric cancer effect of this compound. Cell apoptosis induced by celastrol was detected by using the technique of annexin V-FITC/PI staining and flow cytometry analysis. AGS and BCG-823 cells were treated with celastrol at 1–5 μM for 24 h or 2.5 μM for indicated time points. The results demonstrated that celastrol induced cell apoptosis in a dose- and time-dependent manner. (Figure 3A, 3B) In order to evaluate another character of celastrol, CCK8 assay was applied to analyze cell proliferation. The results indicated the proliferation of AGS and BCG-823 cells was significantly decreased after celastrol administration (Figure 3C).

To investigate CIP2A’s role on cell apoptosis induced by celastrol, protein overexpression technique was applied in the next experimental system. Firstly, we transfected the CIP2A-flag expression construct into AGS and BCG-823 cells for 48 h, followed with or without celastrol for another 6 h, and cell apoptosis was evaluated. As expected, the rate of cell apoptosis induced by celastrol was partly reversed by CIP2A overexpression (Figure 3D). Consistent to our previous study, CIP2A exerts its anti-proliferative and pro-apoptotic effects of celastrol on gastric cancer cells.

### CIP2A mediates cytotoxicity of celastrol by triggering MCL-1 Degradation

**Figure 4:**
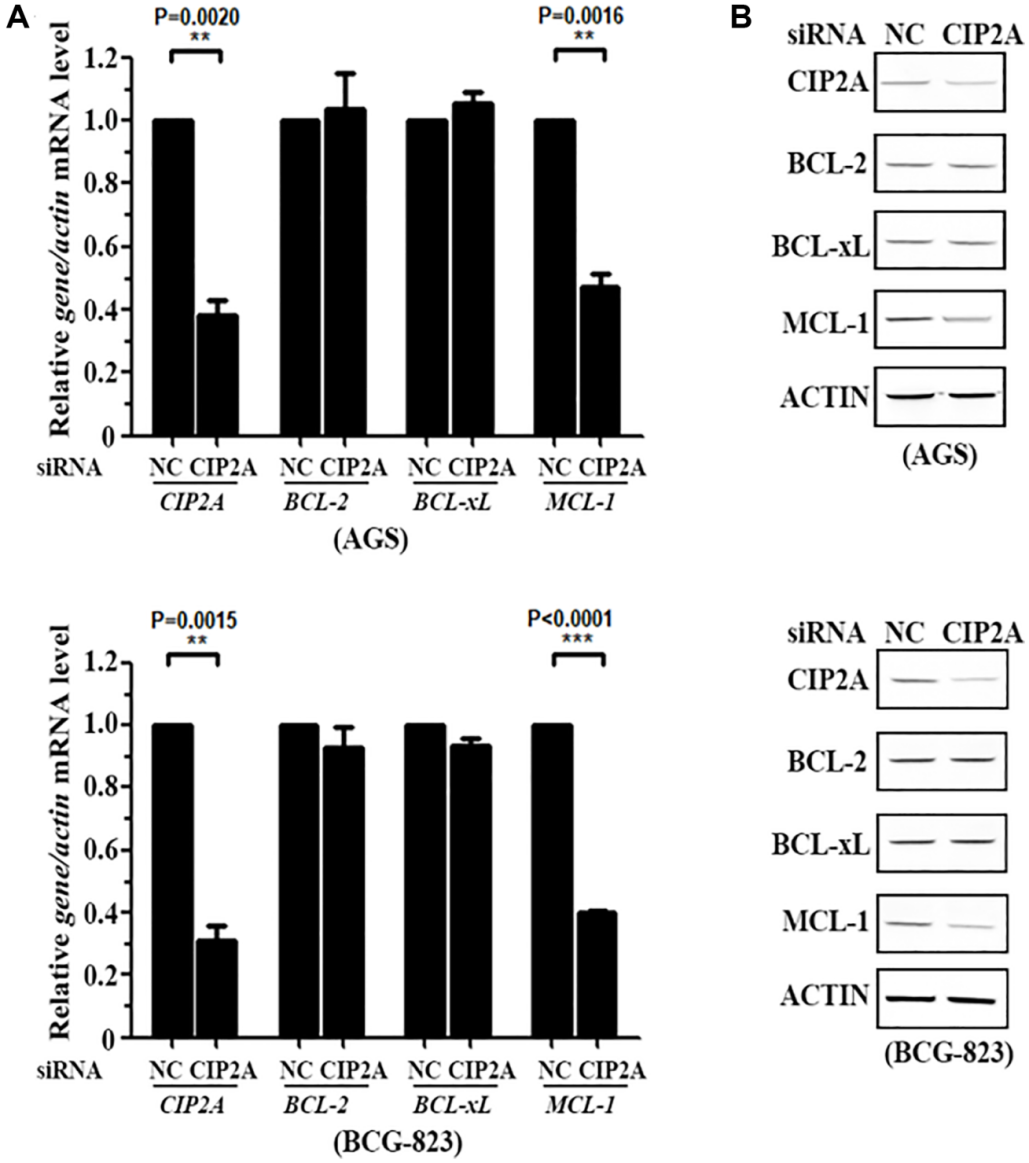
**CIP2A mediates cytotoxicity of celastrol by triggering MCL-1 Degradation.** (**A**, **B**) AGS and BCG-823 cells were transfected with siNC or siCIP2A for 48 h, the expression of CIP2A, BCL-2, BCL-x_L_ and MCL-1 were detected by real-time PCR (**A**) and western blotting (**B**) assays.

Therefore, to further explore whether Myeloid Cell Leukemia 1 (MCL-1), as a downstream molecule of CIP2A signaling pathway, took part in regulating cell apoptosis, we knocked down CIP2A expression level by using RNA interference technology. Results demonstrated silencing of CIP2A specifically decreased MCL-1 expression, but not influenced Bcl-2 and Bcl-x_L_ expression (Figure 4). Our above data confirmed the specificity of CIP2A modulation in cell apoptosis pathway.

### Dephosphorylation by GSK3β enhanced MCL-1 degradation

**Figure 5:**
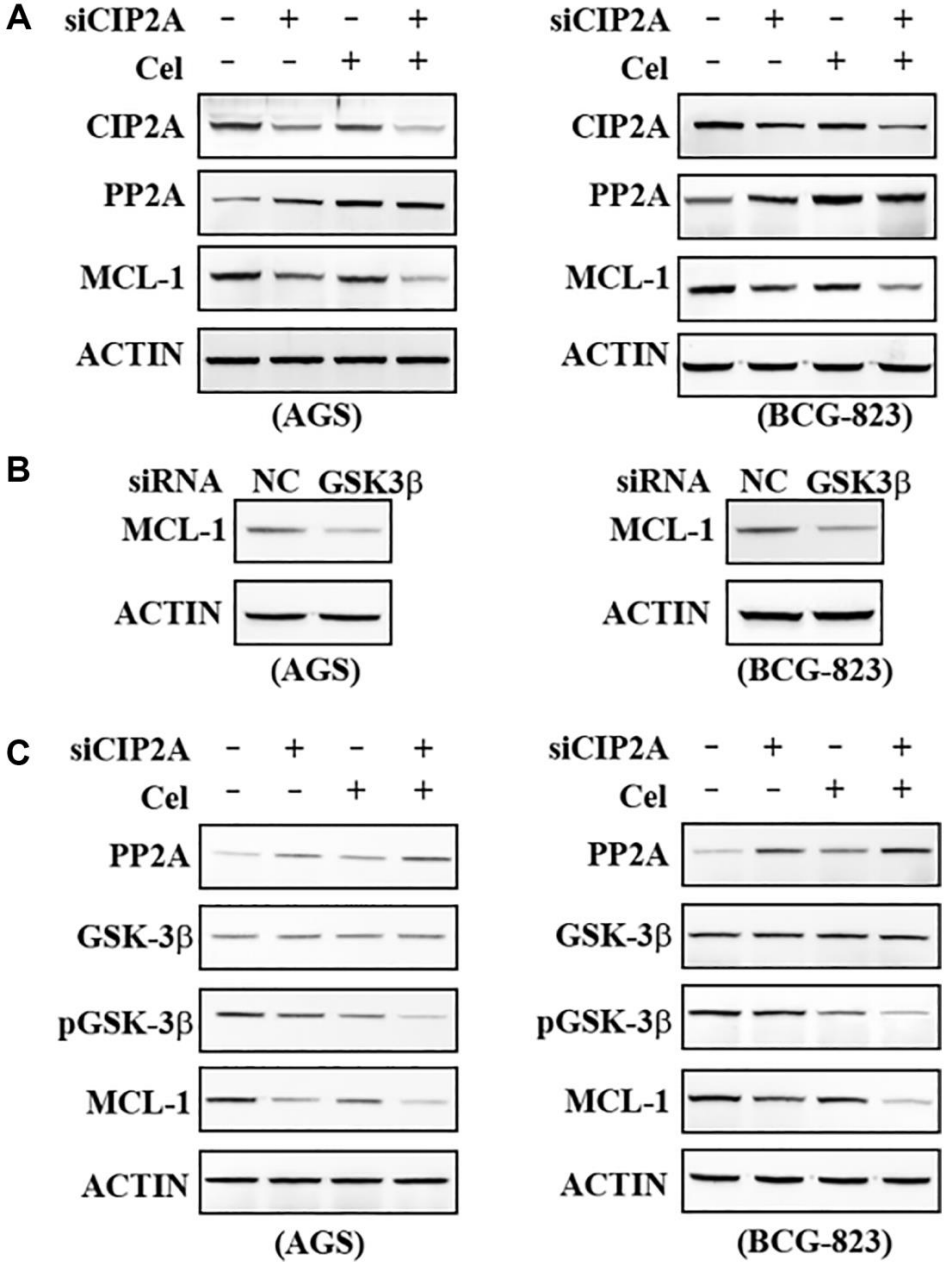
**Dephosphorylation by GSK3β enhanced MCL-1 reduction.** (**A**) AGS and BCG-823 cells were transfected with siNC or siCIP2A for 48 h followed by celastrol (5 μM) for 6 h, lysed, and the lysates were probed with indicated antibodies. (**B**) AGS and BCG-823 cells were transfected with siNC or siGSK3β for 48 h, lysed, and the lysates was probed with anti-MCL-1 antibody. (**C**) AGS and BCG-823 cells were transfected with siNC or siCIP2A for 48 h, followed by celastrol (5 μM) for 6 h, lysed, the expression of PP2A, GSK-3β, pGSK-3β and MCL-1 were detected by Western blot assay.

To identify the signaling pathway(s) mediating the observed apoptosis by celastrol, we firstly detected Protein phosphatase 2A (PP2A) expression in the AGS and BCG-823 cells treated with 5μM celastrol or transfected with siCIP2A. PP2A expression level was upregulated in response to celastrol stimulation and inverse correlation with CIP2A level (Figure 5A). Glycogen synthase kinase 3β (GSK3β) owns a Ser/Thr kinase activity and regulates protein synthesis, cell proliferation, differentiation, motility and apoptosis [22]. We therefore wondered to test whether PP2A regulated GSK3β phosphorylation to influence cell apoptosis. Interestingly, our results showed when GSK3β was silenced, MCL-1 was also downregulated (Figure 5B). Our above data provided the possibility that PP2A-pGSK3β-MCL1 Axis regulated celastrol-triggered apoptosis. To further dissection of this hypothesis, AGS and BCG-823 cells were treated with 5 μM Celastrol or CIP2A-specific knockdown followed by celastrol treatment or not. Results demonstrated PP2A was upregulated and inhibited phosphorylation of GSK3β, and further low level of GSK3β phosphorylation down-regulated MCL-1 expression in response to celastrol-induced degradation of CIP2A (Figure 5C). These results obtained a deeper insight that pro-apoptosis effects of celastrol were involved in GSK3β dephosphorylation activated by PP2A, finally leading to a reduction of pro-survival protein MCL-1.

## DISCUSSION

Gastric cancer is a main contributor to the global burden of disability-adjusted life-years from cancer and accounts for 20% of the total worldwide, following lung and liver cancers, which, respectively, account for 23% and 28% [23]. So far, surgery is still the preferred choice for clinical treatment of gastric cancer, whereas molecular targeted therapy provides an adjuvant strategy for clinical application and improves patient survival and quality of life. Numerous papers have elucidated potential molecular targets, such as immuno-checkpoint blockade, cell apoptosis, epidermal growth factor, angiogenesis, et al. [24]. Therefore, underlying mechanisms of molecular targeted therapies for gastric cancer is important for providing a new strategy of clinical therapy.

CIP2A is involved in sustaining proliferation through preventing cell growth arrest, senescence or differentiation, and its expression level is inversely associated with clinical prognosis of gastric cancer. In our present study, we reported a natural compound celastrol rapidly degraded CIP2A protein at a relatively low concentration (Figure 1B–1E). To our surprise, proteasome inhibitors PS-341 and MG-132 markedly reversed celastrol-induced CIP2A degradation (Figure 2C, 2D), indicating that ubiquitin-proteasome pathway is a main pattern of CIP2A degradation in response to celastrol treatment.

Papers reported celastrol had potent anti-proliferation and pro-apoptosis activity in various cancer cells. Experimental tumor models also confirmed these effects, such as prostate cancer [25], melanoma [26], glioma [27] and NSCLC. However, the mechanism of celastrol anti-gastric cancer effect remains unclear. Here, we demonstrated that celastrol induced apoptosis of gastric cancer cells at a dose- and time-dependent manner in order to exert anti-gastric cancer effect (Figure 3). To test our hypothesis that the anti-cancer effect of celastrol was realized via CIP2A signaling pathway, CIP2A-specific siRNA inference technique was applied to downregulate CIP2A expression. This possibility was confirmed by our data, and these observations clearly indicated CIP2A silencing cells showed the same phenomenon as cells treated with celastrol, and further analysis demonstrated that upon celastrol treatment, knockdown of CIP2A underwent enhanced apoptosis compared with control cells (Figure 3).

Although our studies have indicated celastrol induced cell apoptosis though CIP2A-mediated downstream molecules, the detailed mechanisms involved was not clear. Large number of papers have reported CIP2A was an endogenous inhibitory protein of PP2A. PP2A is a Ser/Thr phosphatase and plays a tumor suppression role in eukaryocytes. Papers have reported Bcl-2 family of proteins regulated cell fate, and the family members exhibited their opposite effects, such as anti-apoptotic or pro-survival activity [28]. Increasing evidences suggested that its family member, Myeloid Cell Leukemia 1 (MCL-1), owned its pro-survival activity in many cancers. Interestingly, celastrol only caused a significant reduction in MCL-1, but not others such as members BCL-2 and BCL-x_L_. In-depth investigation showed that cell apoptosis induced by celastrol was specific modulated by PP2A-GSK3 β-MCL-1 axis, due to PP2A overexpression and then GSK3β dephosphorylation, which in turn acted to diminish MCL-1 protein and ultimately resulted in cell apoptosis (Figure 5). Further analysis indicated PP2A played an early-response sensor in CIP2A ablation triggering cell apoptosis. Downstream of the PP2A complex, GSK3β and MCL-1 mediated cell apoptosis of celastrol. GSK3β dephosphorylation by PP2A furtherly promote MCL-1 degradation. Although PP2A-GSK3β-MCL-1 axis is a main signaling pathway of celastrol-induced apoptosis, the potential contribution of other CIP2A targets did not exclude in coordinating cell apoptosis.

Previous studies has shown celastrol owned many target proteins, such as NF-kB and heat shock response [29]. However, we lacked a clear understanding of the mechanism of celastrol action and direct targets. The aim of our present study was to identify whether celastrol exerted its anti-tumor effect by inducing CIP2A rapidly degradation. The underlying mechanism of celastrol triggered apoptosis was due to PP2A-GSK3β-MCL-1 axis activation followed by degradation of CIP2A, a PP2A suppressor. Increased PP2A activated its downstream GSK3β and terminally led to decreased expression of MCL-1 and eventually resulted in cell apoptosis. In conclusion, celastrol is expected to be applied to the clinical treatment of gastric cancer by targeting an oncoprotein CIP2A. Our work provides further understanding of CIP2A-GSK3β-MCL-1 regulatory axis in tumorigenesis and may present celastrol as a novel therapy for gastric cancer. However, further studies are necessary to confirm the clinical significance of celastrol.
